# Oxygen-Dependent Accumulation of Purine DNA Lesions in Cockayne Syndrome Cells

**DOI:** 10.3390/cells9071671

**Published:** 2020-07-11

**Authors:** Marios G. Krokidis, Mariarosaria D’Errico, Barbara Pascucci, Eleonora Parlanti, Annalisa Masi, Carla Ferreri, Chryssostomos Chatgilialoglu

**Affiliations:** 1 Istituto per la Sintesi Organica e la Fotoreattività, Consiglio Nazionale delle Ricerche, Via P. Gobetti 101, 40129 Bologna, Italy; m.krokidis@inn.demokritos.gr (M.G.K.); annalisa.masi@ic.cnr.it (A.M.); carla.ferreri@isof.cnr.it (C.F.); 2 Institute of Nanoscience and Nanotechnology, N.C.S.R. “Demokritos”, 15310 Agia Paraskevi Attikis, Athens, Greece; 3 Department of Environment and Health, Istituto Superiore di Sanità, Viale Regina Elena 299, 00161 Rome, Italy; mariarosaria.derrico@iss.it (M.D.); barbara.pascucci@ic.cnr.it (B.P.); eleonora.parlanti@iss.it (E.P.); 4 Institute of Crystallography, Consiglio Nazionale delle Ricerche, Monterotondo Stazione, 00015 Rome, Italy; 5 Center for Advanced Technologies, Adam Mickiewicz University, 61-614 Poznań, Poland

**Keywords:** CSA, CSB, oxygen concentration, free radicals, oxidatively generated DNA damage, isotope dilution LC-MS/MS

## Abstract

Cockayne Syndrome (CS) is an autosomal recessive neurodegenerative premature aging disorder associated with defects in nucleotide excision repair (NER). Cells from CS patients, with mutations in *CSA* or *CSB* genes, present elevated levels of reactive oxygen species (ROS) and are defective in the repair of a variety of oxidatively generated DNA lesions. In this study, six purine lesions were ascertained in wild type (wt) CSA, defective CSA, wtCSB and defective CSB-transformed fibroblasts under different oxygen tensions (hyperoxic 21%, physioxic 5% and hypoxic 1%). In particular, the four 5′,8-cyclopurine (cPu) and the two 8-oxo-purine (8-oxo-Pu) lesions were accurately quantified by LC-MS/MS analysis using isotopomeric internal standards after an enzymatic digestion procedure. cPu levels were found comparable to 8-oxo-Pu in all cases (3–6 lesions/10^6^ nucleotides), slightly increasing on going from hyperoxia to physioxia to hypoxia. Moreover, higher levels of four cPu were observed under hypoxia in both CSA and CSB-defective cells as compared to normal counterparts, along with a significant enhancement of 8-oxo-Pu. These findings revealed that exposure to different oxygen tensions induced oxidative DNA damage in CS cells, repairable by NER or base excision repair (BER) pathways. In NER-defective CS patients, these results support the hypothesis that the clinical neurological features might be connected to the accumulation of cPu. Moreover, the elimination of dysfunctional mitochondria in CS cells is associated with a reduction in the oxidative DNA damage.

## 1. Introduction

Hypoxia is a common condition in many diseases. Reduced oxygen supply has been observed during the aging process as well as the onset of neurodegeneration [1]. The hypoxic condition induces proteins involved in the response to oxidative stress, inflammation, apoptosis, mitochondrial metabolism, and autophagy, all processes that play a decisive role in neuronal death [1]. Although it is known that there is a link between hypoxia and neurodegeneration, the mechanisms by which this occurs are yet to be determined. It has been reported that hypoxia is associated with the familial and sporadic Alzheimer disease [2,3]. In particular, hypoxia can increase Aβ production [4], enhance tau phosphorylation [5], induce neuroinflammation [6], increase reactive oxygen species generation [2], and elicit abnormal mitochondrial function [7]. Moreover, enhancing α-synuclein expression and aggregation, hypoxia is a potential cause of Parkinson disease [8] and it is a causative factor of both the onset and progression of amyotrophic lateral sclerosis [9]. In addition, a role of hypoxia in multiple sclerosis is also observed [10].

**Figure A1:**
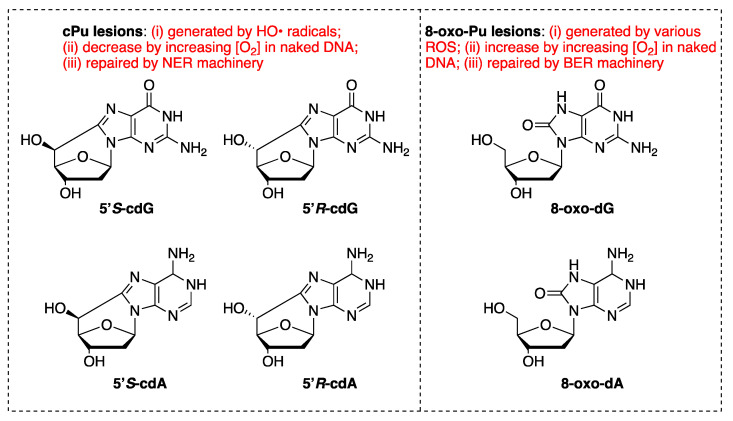
Left: Structures of 5′,8-cyclo-2′-deoxyguanosine (cdG) and 5′,8-cyclo-2′-deoxy-adenosine (cdA) in their 5′*R* and 5′*S* diastereomeric forms; Right: Structure of 8-oxo-2′-deoxyguanosine (8-oxo-dG) and 8-oxo-2′-deoxyadenosine (8-oxo-dA).

Cockayne syndrome (CS) is a multi-system disorder associated in many patients with defects in nucleotide excision repair (NER), a process that removes a variety of DNA-blocking lesions [11]. CS is cancer-free and the clinical features are pre- or post-natal growth failure, leading to a characteristic appearance of so-called cachectic dwarfism, premature aging and progressive neurological dysfunction. Over 90% of CS cases are due to mutations in either the *CSA* or *CSB* genes. CS cells are defective in the removal of lesions in the transcribed strand of actively transcribed genes (transcription-coupled NER sub-pathway, TCR) [11]. Although CSA and CSB patients present similar clinical features, the proteins have different activity and functions in the TCR. An important clue in understanding the severe neurological abnormalities of CS was the discovery that the defect in TCR is not limited to lesions repaired by NER, but extends to oxidative DNA lesions that are a substrate for base excision repair (BER) [12]. The sensitivity of CS cells to oxidatively-generated DNA damage indicates that these proteins play a role in the removal of oxidative DNA damage [13,14,15]. In particular, it has been shown that CSA protein in untreated human fibroblasts controls the steady state level of 8-oxo-7,8-dihydro-2′-deoxyguanosine (8-oxo-dG) and that keratinocytes from CSA patients accumulated (5′*S*)-5′,8-cyclo-2′-deoxyadenosine (5′*S*-cdA) under standard atmospheric oxygen tension (cf. Appendix A Figure A1 for the structure of the lesions) [14]. Accumulation of 5′*S*-cdA in organs of *CSB* knockout mice has also been reported [16]. Furthermore, the CSB protein has been shown to stimulate APE1 activity and to protect against agents that induce BER intermediates [17,18]. Recent findings have shown that CS proteins are involved in the recognition, signaling and processing of single-strand breaks (SSBs) as well as double-strand breaks (DSBs), relevant lesions in neurodegenerative disorders [19]. Moreover, high levels of mitochondrial DNA damage, as well as a hypersensitivity to bioenergetic inhibitors and an altered mitochondrial respiratory chain, have been reported in CSB mouse cells [20]. It was also demonstrated that human CS cells present an altered redox balance and mitochondrial dysfunction [21,22,23]. Furthermore, it was reported that, by overexpressing Parkin, a protein involved in mitophagy, CSA cells successfully recovered from mitochondrial dysfunction and were protected from apoptosis [24]. Finally, very recent data showed that CS proteins play an important role in protecting cells from senescence [25,26].

**Figure A2:**
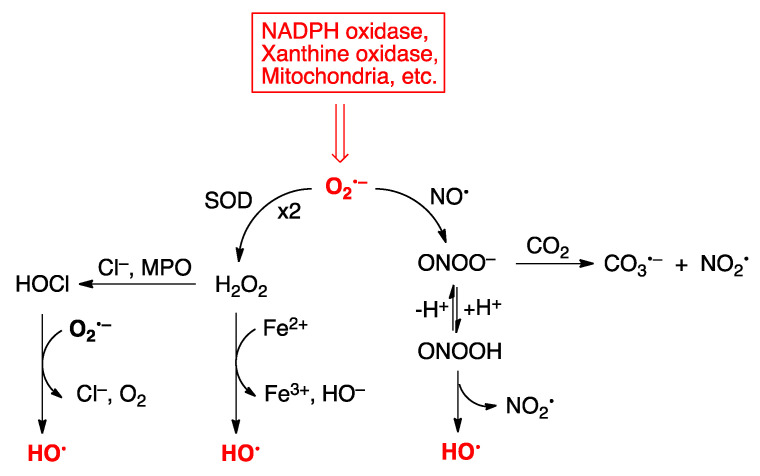
The main processes that generate HO^•^ radicals in living systems are the Fenton reaction of H_2_O_2_, the reduction in HOCl by superoxide radical anion (MPO = myeloperoxidase) and the spontaneous decomposition of protonated ONOO^−^, respectively.

Reactive oxygen species (ROS) are formed during normal metabolism in pathophysiological processes as well as by UV light or ionizing radiation. These species are very reactive towards DNA, proteins, and lipids, and can trigger various illnesses and enhance aging processes. Among ROS, the hydroxyl radical (HO^•^) is the most reactive species (cf. Appendix A Figure A2) [27,28,29]. Purine 5′,8-cyclo-2′-deoxynucleosides (short name: 5′,8-cyclopurine; acronym: cPu) are well recognized products of nucleic acid damage [30,31,32,33,34]. 5′,8-Cyclo-2′-deoxyadenosine (cdA) and 5′,8-cyclo-2′-deoxyguanosine (cdG), existing as 5′*R* and 5′*S* diastereoisomeric forms (Figure A1), are generated by the reaction of HO^•^ radicals with genetic material via C5′ radical chemistry of purine moieties [34] and are stable in acidic conditions [35,36]. Their formation is inversely correlated to oxygen concentration [29,37,38] and they are substrates of NER [39,40]. On the other hand, the well-known 8-oxo-7,8-dihydro-2′-deoxypurines (8-oxo-dG and 8-oxo-dA in Figure A1) are generated by oxidation at the C8 position by a variety of ROS, like HO^•^ and ROO^•^ radicals, H_2_O_2_, singlet oxygen or ONOO^−^ and are labile in acidic conditions [36,41]. The fact that cPu DNA lesions do not suffer from stability issues and artifacts like other oxidatively generated DNA lesions, along with their specific generation by HO^•^ radicals and their exclusive repair by NER, allows them to be considered excellent candidate markers of DNA damage.

In the present work we considered DNA damage of purine bases in CSA and CSB-transformed fibroblasts grown under various oxygen levels. In particular, we adjusted the atmosphere to have 21%, 5% and 1% of O_2_ referred to hyperoxia, physioxia and hypoxia conditions, respectively [42,43]. In vivo, mammalian cells reside in an environment of 0.5 –10% O_2_ (depending on the tissue location within the body), whilst standard in vitro cell culture is carried out under the atmospheric oxygen tension of 21% (what we termed hyperoxia) [44]. Our study contributes to a better understanding of the role of oxygen incubation conditions and deepens the comprehension of DNA damage when cellular repair capacity is affected. Here, we show that CSA and CSB-defective cells present higher basal levels of the six purine lesions than controls, measured with a very sensitive protocol (LC-ESI-MS/MS system with isotopomeric internal standards) [33,45] and these results may be relevant to explain the clinical outcome of this disease. Furthermore, the analysis of DNA damage was also carried out in CSA-defective cells overexpressing Parkin.

## 2. Materials and Methods

### 2.1. Cell Lines

CSA and CSB SV40-transformed cell lines were established and cultured as previously described [46]. More precisely, an isogenic cell line that expresses the wtCSA protein tagged with the Flag and HA epitopes (CS3BE-wtCSA) was used (manuscript in preparation, Lanzafame M. et al.). The defective counterpart is CS3BE [24]. For CSB cell lines we used CS1AN-wtCSB and CS1AN [21]. Defective cell lines carry the empty vector. Cell culture studies are grown under standard atmospheric oxygen tension, 21% O_2_ (hyperoxia), 5% O_2_ (physioxia) and 1% O_2_ (hypoxia). Cells overexpressing Parkin were generated as indicated in Pascucci et al. [24].

### 2.2. Enzymatic Digestion and Quantification of Modified Nucleosides by Stable Isotope LC-MS/MS

Cells were lysed, genomic DNA was isolated using a high-salt extraction procedure [45,46] and lesions levels were quantified as described previously [33,36,45,47,48]. Briefly, 10 μg of DNA were enzymatically digested in a reaction mixture including 0.2 mM pentostatin, 5 μM BHT, 3 mM deferoxamine and the internal standards ([^15^N_5_]-5′*S*-cdA, [^15^N_5_]-5′*R*-cdA, [^15^N_5_]-5′*S*-cdG, [^15^N_5_]-5′*R*-cdG, [^15^N_5_]-8-oxo-dG and [^15^N_5_]-8-oxo-dA), the samples were filtered off by centrifugation through a 3 kDa microspin filter, cleaned up and enriched by an HPLC-UV system coupled with a sample collector and injected to the LC-MS/MS system. The quantification of the modified nucleosides was carried out by a triple-stage quadrupole mass spectrometer using positive electrospray ionization (ESI) following a gradient program (2 mM ammonium formate, acetonitrile and methanol) and the detection was executed in multiple reaction monitoring mode (MRM) using the two most intense and characteristic precursor/product ion transitions for each lesion (Table S1) [29,34].

### 2.3. Statistical Analysis

All measurements were performed in triplicate and the data were expressed as mean ± standard deviation (SD). The unpaired t-test was used for statistical analysis and a two-tailed *p*-value < 0.05 and *p*-value < 0.01 were considered to indicate a statistical significant difference.

## 3. Results

### 3.1. Purine DNA Lesions Levels in wtCSB and Defective CSB Cells

**Figure 1:**
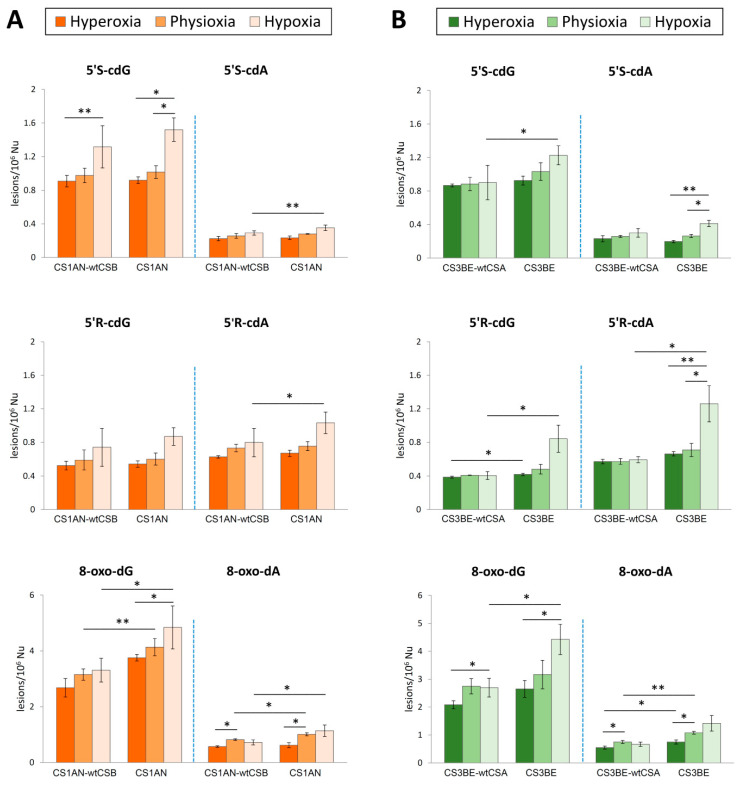
The levels (lesions/10^6^ Nu) of 5′*S*-cdG, 5′*S*-cdA, 5′*R*-cdG, 5′*R*-cdA, 8-oxo-dG and 8-oxo-dA measures by LC-MS/MS. (**A**) Purine DNA lesions in DNA samples isolated from CS1AN-wtCSB and CS1AN cells in hyperoxic, physioxic and hypoxic conditions (cf. Tables S2 and S4 for details). (**B**) Purine DNA lesions in DNA samples isolated from CS3BE-wtCSA and CS3BE cells in hyperoxic, physioxic and hypoxic conditions (cf. Tables S3 and S5 for details). The error bars represent the standard deviation of the mean, calculated from three independent samples, * denotes a statistically significant difference (*p* < 0.05) between the groups, ** denotes a statistically significant difference (*p* < 0.01) between the groups.

Genomic DNA of both CS1AN-wtCSB (wtCSB) and CS1AN (defective CSB) cells cultivated under hypoxic conditions (1%) compared with physioxic (5%) and hyperoxic conditions (21%) was isolated from cellular samples, hydrolyzed to single nucleosides by an enzymatic cocktail containing nucleases, and analyzed by liquid chromatography with tandem mass spectrometry (LC–MS/MS) for the determination of the modified nucleosides (the four cPu and the two 8-oxo-Pu), in accordance to a recently optimized protocol [33,36,45]. Figure 1A (see also Tables S2 and S4 in Supporting Material) shows significantly increased levels of cPu and 8-oxo-Pu in wtCSB and defective CSB cell lines under hypoxia (light orange bars) compared with physioxia and hyperoxia (middle and dark orange, respectively).

Focusing on the effects of distinct oxygen tension on base damage, 8-oxo-dG was the most intense among the six purine lesions varying oxygen incubation conditions, found with levels of 2.37–3.78/10^6^ nucleosides (Nu) in wtCSB cells and 3.63–5.66/10^6^ Nu in defective ones. Among the four cPu lesions, the 5′*S*-cdG was indicated with levels of 0.84–1.57/10^6^ Nu and 0.89–1.65/10^6^Nu in wtCSB and defective cells, respectively.

In CS1AN-wtCSB cells, statistically increased levels of 5′*S*-cdG damage were found in hypoxic conditions compared to hyperoxia (*p* = 0.005) and increased levels of 8-oxo-dA were observed in physioxia conditions as compared to hyperoxia (*p* = 0.017).

On the other hand, in mutant CS1AN cell line significant enhancement of 5′*S*-cdG was exhibited under hypoxia compared to hyperoxia (*p* = 0.012) and to physioxia conditions (*p* = 0.032). Furthermore, 8-oxo-dG was found significantly raised under hypoxia compared to hyperoxia (*p* = 0.01) and 8-oxo-dA under physioxic as compared to hyperoxic conditions (*p* = 0.044).

Statistical significance was observed in the increased values of 5′R-cdA (*p* = 0.027), 5′S-cdA (*p* = 0.003), 8-oxo-dG (*p* = 0.018) and 8-oxo-dA (*p* = 0.028) in defective CS1AN cells under hypoxic conditions compared to the wild type counterpart (light orange bars in Figure 1A). Lastly, differences in the levels of 8-oxo-dG and 8-oxo-dA were detected under physioxia (*p* = 0.005 and *p* = 0.011) between wild type and defective cell lines (middle orange bars in Figure 1A).

### 3.2. Purine DNA Lesions Levels in wtCSA and Defective CSA Cells

Alterations in the levels of purine DNA lesions were also observed in the CS3BE-wtCSA (wtCSA) and CS3BE (defective CSA) cells under the three distinct experimental conditions as highlighted in Figure 1B. All the experimental values and the statistical analysis are collected in Supporting Material (Tables S3 and S5). In normal cells, a statistically significant increased accumulation of 8-oxo-dG occurred under hypoxia compared to hyperoxia (*p* = 0.044) as found also for 8-oxo-dA under physioxia compared to hyperoxic conditions (*p* = 0.047).

In defective CSA cells, significantly accumulated levels of 8-oxo-dA were observed in physioxia compared to hyperoxia (*p* = 0.042, dark vs. middle green bars). Moreover, the levels of 8-oxo-dG were found elevated under hypoxic as compared to hyperoxic conditions (*p* = 0.028, dark vs. light green bars), and statistically significant alterations were observed in the levels of 5′S-cdA under hypoxia as compared to hyperoxia (*p* = 0.006) as well as under physioxic compared to hypoxic conditions (*p* = 0.02). Regarding 5′*R*-cdA, statistically significant increased levels were measured in defective cells under hypoxia as compared to physiological (*p* = 0.033) and hyperoxia conditions (*p* = 0.038).

Among wild type and defective cells, statistically higher levels of 5′*R*-cdG and 8-oxo-dA occurred in mutant as compared to normal cells in hyperoxic conditions (*p* = 0.037 and *p* = 0.04, respectively; dark green bars in Figure 1B). 8-oxo-dA was also found accumulated under physioxia between CS3BE-wtCSA and CS3BE cells (*p* = 0.003). Under low oxygen concentration (1%), significantly increased levels of 5′*S*-cdG (*p* = 0.049), 5′*R*-cdG (*p* = 0.047), 5′*R*-cdA (*p* = 0.045) and 8-oxo-dG (*p* = 0.044) were found in CSA-defective cells as compared to normal cell lines (light green bars in Figure 1B).

All together, these data clearly indicate that the accumulation of oxidative lesions in the defective cell lines is due to the role of the CSA and CSB proteins in their repair of this oxidatively induced DNA damage, while normal counterparts in the same conditions have a normal repair capacity.

### 3.3. Diastereoisomeric Ratio in cPu Lesions

The 5′R/5′S ratios, which can provide an important indication on structural conformation of both diastereoisomers in association with their abundance and repair, were found to be similar for cdG and cdA lesions in CS1AN-wtCSB and CS1AN cell lines under the three distinct experimental conditions, with a ratio of cdA approximately 5-fold higher than the cdG values (Table 1 and Table S6). On the other hand, the diastereomeric ratio showed a similar trend for cdG only in CS3BE-wtCSA regardless of oxygen tension, and higher levels in CS3BE cells under hypoxia (approximately 1.5-fold). Furthermore, a slight decrease in the 5′R/5′S ratio was observed for cdA lesions going from hyperoxia to physioxia or hypoxia conditions, both for wild type and deficient cells. It should also be highlighted there are higher levels of 5′R/5′S ratio for cdA in deficient cells compared to the wild type cell line under the three experimental conditions, evidence that was not observed in CS1AN-wtCSB and CS1AN cells.

### 3.4. Comparison of cPu and 8-oxo-Pu Levels

**Figure 2:**
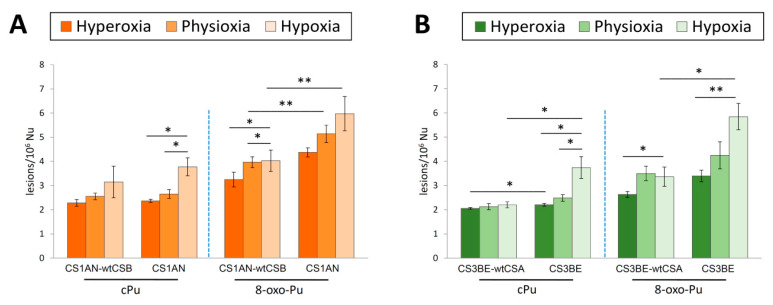
Comparison between cPu and 8-oxo-Pu lesions under hyperoxia, physioxia and hypoxia. (**A**) The levels (lesions/10^6^ Nu) of cPu and 8-oxo-Pu lesions in genomic DNA extracted from CS1AN-wtCSB and CS1AN cells. (**B**) The levels (lesions/10^6^ Nu) of cPu and 8-oxo-Pu lesions in genomic DNA extracted from CS3BE-wtCSA and CS3BE cells. The error bars represent the standard deviation of the mean, calculated from three independent samples, * denotes a statistically significant difference (*p* < 0.05) between the groups, ** denotes a statistically significant difference (*p* < 0.01) between the groups.

Comparing the accumulation of total 8-oxo-Pu and cPu, a 1.3–1.9-fold higher level for the first one was observed in CS1AN-wtCSB and CS1AN cells, as shown in Figure 2A (see Tables S7 and S9). In CS1AN-wtCSB cells, statistically increased 8-oxo-Pu levels were detected under physioxic and hypoxic as compared to hyperoxic conditions (*p* = 0.035 and *p* = 0.027). There is also an increase in 8-oxo-Pu in CS1AN in hypoxia as compared to physioxia and hyperoxia, although there are no statistically significant differences.

In CS1AN cells, total cPu were found to have significantly increased levels comparing hyperoxia to hypoxia (*p* = 0.023) and physioxia to hypoxia (*p* = 0.043). Among wild type and CSB-defective cells, significantly higher levels of 8-oxo-Pu were found under physioxia and hypoxia conditions (*p* = 0.006 and *p* = 0.007, respectively).

Total 8-oxo-Pu levels were observed approximately 1.3–1.7-fold higher compared to total cPu in the CS3BE-wtCSA and the CS3BE cells as depicted in Figure 2B and summarized in Tables S8 and S10. In normal cell line, statistically significant enhancement of 8-oxo-Pu was depicted in hypoxic as compared to hyperoxic conditions (*p* = 0.044). Total 8-oxo-Pu was also found to be statistically elevated in defective CSA cell lines under similar oxygen tension (*p* = 0.007). Emphasis should be given to the significant increase in cPu in the CS3BE cells under hyperoxia and physioxia as compared to hypoxia (*p* = 0.034 and *p* = 0.028, respectively).

Under hyperoxic conditions, elevated levels of total cPu were found in the defective CSA cell line as compared to normal cells (*p* = 0.035). Under hypoxia, the levels of total cPu and 8-oxo-Pu were observed as being significantly higher in CS3BE cells compared to wild type (*p* = 0.016 and *p* = 0.041, respectively).

### 3.5. Purine DNA Lesions Levels in Defective CSA Cells Overexpressing Parkin

In order to test if mitochondria, by ROS production, have a role in the accumulation of oxidative DNA damage, we measured the amount of DNA damage also in CSA cells overexpressing Parkin. Parkin is a protein involved in mitophagy, a mechanism devoted to the removal of dysfunctional mitochondria. In our previous paper we showed that overexpression of this protein in CSA cells, that are characterized by a mitochondrial dysfunction, eliminates the damaged mitochondria and reverts the CSA phenotype [24].

**Figure 3:**
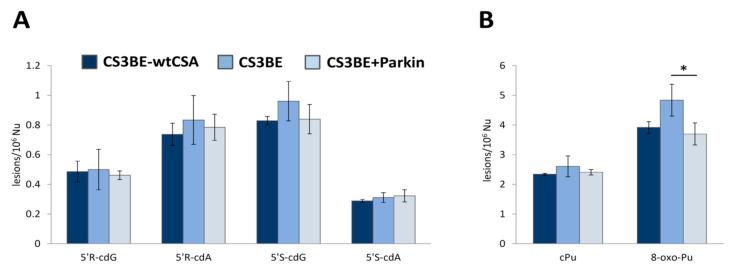
Purine DNA lesions measured by LC-MS/MS. (**A**) The levels (lesions/10^6^ Nu) of 5′R-cdG, 5′S-cdG, 5′R-cdA and 5′S-cdA in DNA samples isolated from CS3BE-wtCSA, CS3BE and CS3BE + Parkin cells. (**B**) The levels (lesions/10^6^ Nu) of total cPu and 8-oxo-Pu lesions in genomic DNA extracted from CS3BE-wtCSA, CS3BE and CS3BE + Parkin cells. The error bars represent the standard deviation of the mean, calculated from three independent samples. * denotes a statistically significant difference (*p* < 0.05) between the groups.

The measurement, in hyperoxia conditions, of the levels of cPu indicated a slight accumulation of these lesions in defective cells as compared to normal ones, as already reported in the previous paragraph, but in CSA cells overexpressing Parkin (CS3BE + Parkin) a reduction in the levels of cPu was observed, although is not statistically significant (see Figure 3 and Tables S11 and S12 for detailed values and statistical analysis). As depicted in Figure 3A, 5′S-cdG was found the most predominant among the four cPu lesions with detected levels of 0.80–0.85/10^6^ nucleosides in CS3BE-wtCSA cells, 0.87–1.11/10^6^ Nu in defective ones and 0.73–0.90/10^6^ Nu in CS3BE + Parkin cells. Regarding the diastereoisomeric outcome, in all cases the 5′R/5′S ratio is almost the same for cdG and cdA lesions, cdA being approximately 4.5-fold higher than the corresponding value of cdG. Total 8-oxo-Pu levels were found approximately 1.7-fold more elevated compared with total cPu in CS3BE-wtCSA cells, 1.9-fold in CS3BE cells and 1.5-fold in CS3BE + Parkin ones, as depicted in Figure 3B (Table S13). CS3BE + Parkin cells accumulated significantly lower levels of 8-oxo-Pu compared to defective CS3BE cells, reaching values observed in normal cells.

## 4. Discussion

In this study we provide for the first time the connection between oxygen concentration and DNA damage induction in DNA repair-defective CSA and CSB cells. Several lines of evidence support the role of oxidative DNA damage in neurodegenerative diseases and in aging processes. The human disorder CS involves a defect in DNA repair and transcription and presents as clinical features, neurological and developmental abnormalities and premature ageing. Our aim was to elucidate the role of oxygen incubation conditions when cellular repair capacity is affected.

The accumulation of specific purine DNA lesions such as cPu, selective substrates of NER cellular machinery and direct markers of HO^•^ radical damage, was followed-up together with 8-oxo-Pu which derive from different ROS and can be repaired by BER. Standard oxygen incubation conditions (21% O_2_) were used. Often 21% oxygen is erroneously referred as “normoxic” incubation condition; in this study we evaluated this condition as “hyperoxia”, considering that the amount of oxygen that the cell receives is substantially higher than the in vivo exposure [43,49]. An isotope–dilution LC-MS/MS methodology was followed for the accurate quantification of the DNA adducts, increasing the reliability of the process and enhancing to a great extent the characteristics of reproducibility and recovery of the analytical protocol. Herein, it should be emphasized that the presence of argon, chelating agents and antioxidants, during the digestion steps, are parameters that are of the utmost importance for avoiding workup artifacts caused by the oxygen influence on the nucleoside moiety. Subsequently, the accurate quantification of DNA adducts was carried out by varying oxygen concentration, using 5% O_2_ (physioxia) and 1% O_2_ (hypoxia).

Hypoxia (1% O_2_) is known to induce a rapid increase in the expression of several genes, including inducible nitric oxide synthase (iNOS) [50], to activate the xanthine oxidase pathway [51], to increase catecholamine production [52] and to increase the rate of electron leakage within the mitochondria [53]. All of these factors subsequently induce increased levels of intracellular ROS. Under these conditions a wide variety of biomolecules, including nucleic acids, are damaged, but also the activity of the DNA repair machinery is inhibited causing DNA damage accumulation [54,55]. A positive correlation between the increase in the iNOS expression levels and the accumulation of DNA damage has been reported [50,56,57]. It is well known that iNOS protein is responsible for the production of cellular nitric oxide (NO^•^), which, reacting with superoxide radical anion (O_2_^•–^), forms peroxynitrite (ONOO^−^) (see Appendix A Figure A2) [28]. ONOO^−^ is able to directly generate 8-oxo-Pu lesions, or, associated with its protonated form, decomposes spontaneously forming HO^•^ and ^•^NO_2_ radicals. The HO^•^ radical can directly react with the DNA, either by hydrogen abstraction from the sugar, leading also to cPu lesions, or by addition/hydrogen abstraction involving the base moieties, forming a variety of products, including the 8-oxo-Pu lesions. CS cells express high levels of iNOS protein and present an accumulation of peroxynitrite [58].

Two important observations in our study are (i) the relatively small differences in the levels of cPu vs. 8-oxo-Pu, (ii) increased levels of cPu and 8-oxo-Pu lesions going from hyperoxia to physioxia to hypoxia for CSA and CSB cells (Figure 2). The same trends are observed for the individual six lesions (Figure 1). Previous studies carried out in calf-thymus DNA in vitro showed that (i) cPu are induced at much lower levels than 8-oxo-Pu when HO^•^ radicals are generated by Fenton-type reaction [59], and (ii) the cPu and 8-oxo-Pu gradually decrease and increase, respectively, by increasing oxygen concentration, reaching a gap of ~130 times at 15% of O_2_ when HO^•^ radicals are generated by ionizing radiations of aqueous solutions [29,38]. The abundance of cPu lesions in the present study suggests their potential role as candidate markers for pathologies with defects of NER like CS.

In our experiments, increased levels of cPu and 8-oxo-Pu lesions were observed in CSA-defective cells (CS3BE) as compared to wtCSA cells (CS3BE-wtCSA) under various oxygen tensions, with a significant enhancement under hypoxia conditions (Figure 1B). A similar trend was also observed in defective CSB (CS1AN) cells compared to normal fibroblasts (Figure 1A). The observed accumulation of cPu under hypoxic conditions, suggests that CS proteins are involved in the cPu repair by NER. Recently, we published that also another DNA repair-defective syndrome, XPA, presents an accumulation of cPu lesions under hypoxia conditions [60]. It is well known that these adducts are transcription-blocking lesions and the accumulation of this type of oxidative DNA damage in actively transcribed genes has been associated with neuronal death [61]. All these data support the notion that, in addition to the defect in TCR, repair of lesions in the overall genome might be affected in CS cells [62]. It is well known that the neurological defects observed in CS patients are radically different from those in XPA patients, characterized by extensive neurodegeneration. In fact, in such patients, neurodevelopment is the main clinical feature while neurodegeneration is confined to Purkinje cells [63]. Therefore, the accumulation of cPu plays a fundamental role in the neurological component of these two syndromes. Moreover, we observed, in hypoxia conditions, the accumulation of 8-oxo-Pu, lesions repaired by the BER pathway, confirming the role played by CS proteins in the repair of oxidative lesions [14].

Furthermore, the amount of purine DNA lesions was measured in CSA cells overexpressing Parkin (CS3BE + Parkin) in hyperoxia conditions. It is interesting to note that in CSA cells overexpressing Parkin, a protein involved in mitophagy, all measured lesions were less than those measured in the CSA cells, and approached the levels of the wt cells (CS3BE-wtCSA), although without statistical significance. These results are in agreement with the role of mitochondrial dysfunction in the pathogenesis of CS. In fact, the overexpression of Parkin, eliminating the damaged mitochondria, reverts the CSA phenotype [24]. The elimination of mitochondria, and so the primary source of ROS, via overexpression of Parkin, leads to a reduction in the oxidative induced lesions.

Additionally, the 5′*S* diastereomers of cdG were found to be nearly twice those of their 5′*R* counterpart whereas the opposite is observed for cdA, being 5′*R* diastereomers 2–3 times higher than their 5′*S* counterpart (Table 1) and independent of oxygen tension. The role of the diastereoisomeric ratios 5′*R*/5′*S* in cPu is not yet fully understood. There are at least two factors playing a role: (i) the more efficient repair of the 5′*R* diastereomers of the cPu reported in human cell extracts by NER [40], and (ii) the structural characteristics of the transition states associated with the C5′ radical cyclization that generates the cPu lesions [29]. In our recent work on inflamed IBD-colon biopsies versus their normal counterpart, we found the 5′*S* diastereomers of cdA and cdG are at higher levels than their 5′*R* counterparts, supporting the more efficient repair of the 5′*R* diastereomers of the cPu reported in human cell extracts [57]. On the other hand, in the present study we measured the 5′*R*/5′*S* ratio to be identical for wtCSB and defective CSB (being 0.58–0.59 for cdG and 2.82–2.84 for cdA), which is in agreement with the recent published values for EUE-pBD650 (wt) and EUE-siXPA (deficient) human embryonic epithelial cell lines (0.29–0.32 for cdG and 2.69–2.94 for cdA and independent of oxygen tension) [60]. We believe that the measured values of the 5′*R*/5′*S* ratio for cPu lesions for CSA, CSB and XPA reflect important multi-component parameters connected to neurodegenerative diseases or pathologies with deficiencies of the NER process.

## 5. Conclusions

In this work, the abundance of an important family of oxidatively induced DNA lesions was investigated in DNA repair-deficient cell lines such as CSA and CSB, cultured under various oxygen conditions. The accumulation of cPu, which are the smallest tandem purine lesions and exclusive (or unique) markers of HO^•^ damage, and comparison with 8-oxo-Pu, characteristic lesions arising by various ROS, are detailed. Important differences are depicted among the two distinct cell types. cPu formation strongly occurs as the oxygen concentration rises in CSA- and CSB-deficient cell lines. Taking into account that the cPu DNA lesions do not suffer from stability issues and artifacts of other oxidatively generated DNA lesions, these results revealed the use of cPu lesions as reliable markers of the oxygen tension under NER-defective conditions. Moreover, these findings give strength to the hypothesis that defective repair of oxidative DNA damage, in particular the cPu, is involved in the clinical neurological features of CS patients.

## Figures and Tables

**Table 1:** Total amount of 5′*R*/5′*S* ratio of cPu lesions in DNA isolated from CS1AN-wtCSB and CS1AN cells and CS3BE-wtCSA and CS3BE cells.

Samples	cdG	cdA
	5′*R*/5′*S*	5′*R*/5′*S*
CS1ANwtCS-B	0.58 ± 0.03	2.82 ± 0.08
CS1AN	0.59 ± 0.01	2.84 ± 0.13
CS3BE-wtCSA	0.46 ± 0.01	2.26 ± 0.26
CS3BE	0.54 ± 0.13	3.06 ± 0.35

## Supplementary Materials

The following are available online at https://www.mdpi.com/2073-4409/9/7/1671/s1, Figure S1: Protein expression analysis for CSA, CSB and Parkin, Table S1: MRM transitions, Table S2: The levels of lesions in genomic DNA extracted from CS1AN-wtCSB and CS1AN cells, Table S3: The levels lesions in DNA samples isolated from CS3BE-wtCSA and CS3BEcells, Table S4: Values of t-Test in DNA samples isolated from CS1AN-wtCSB and CS1AN cells, Table S5: Values of t-Test in DNA samples isolated from CS3BE-wtCSA and CS3BE cells in each condition, Table S6: 5′R/5′S ratio of cPu lesions in genomic DNA extracted from CS1AN-wtCSB and CS1AN cells and CS3BE-wtCSA and CS3BE cells, Tables S7 and S8: Total amount of cPu and 8-oxo-Pu lesions in DNA isolated from CS1ANwtCSB and CS1AN cells and CS3BEwtCSA and CS3BE cells, Tables S9 and S10: Values of t-Test in DNA isolated from CS1AN-wtCSB and CS1AN cells and CS3BE-wtCSA and CS3BE cells, Table S11: The levels lesions in DNA samples extracted from CS3BE-wtCSA, CS3BE and CS3BE + Parkin cells, Table S12: Values of t-Test, Table S13: Total amount of cPu and 8-oxo-Pu lesions in DNA isolated from CS3BE-wtCSA, CS3BE and CS3BE + Parkin cells.
