# Correction: Krokidis, M.G., et al. Oxygen-Dependent Accumulation of Purine DNA Lesions in Cockayne Syndrome Cells. *Cells* 2020, *9*, 1671

**DOI:** 10.3390/cells10010041

**Published:** 2020-12-30

**Authors:** Marios G. Krokidis, Mariarosaria D’Errico, Barbara Pascucci, Eleonora Parlanti, Annalisa Masi, Carla Ferreri, Chryssostomos Chatgilialoglu

**Affiliations:** 1 Istituto per la Sintesi Organica e la Fotoreattività, Consiglio Nazionale delle Ricerche, Via P. Gobetti 101, 40129 Bologna, Italy; m.krokidis@inn.demokritos.gr (M.G.K.); annalisa.masi@ic.cnr.it (A.M.); carla.ferreri@isof.cnr.it (C.F.); 2 Institute of Nanoscience and Nanotechnology, N.C.S.R. “Demokritos”, Agia Paraskevi Attikis, 15310 Athens, Greece; 3 Department of Environment and Health, Istituto Superiore di Sanità, Viale Regina Elena 299, 00161 Rome, Italy; mariarosaria.derrico@iss.it (M.D.); barbara.pascucci@ic.cnr.it (B.P.); eleonora.parlanti@iss.it (E.P.); 4 Institute of Crystallography, Consiglio Nazionale delle Ricerche, Monterotondo Stazione, 00015 Rome, Italy; 5 Center for Advanced Technologies, Adam Mickiewicz University, 61-614 Poznań, Poland

The originally published article [1] contains an error in the depiction of the structures of adenine derivatives in Figure A1 (Appendix A).

The correct structures are shown below.


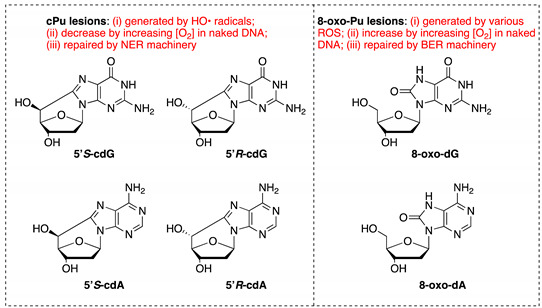


The authors would like to apologize for any inconvenience this has caused the readers. The change does not affect the scientific results.
