# Supplementary material for: Oxygen-Dependent Accumulation of Purine DNA Lesions in Cockayne Syndrome Cells
Source: Cells. 2020 Jul 11;9(7):1671. doi: 10.3390/cells9071671 (PMC7407219; doi:10.3390/cells9071671)
Supplement: Supplementary file 1 [file cells-09-01671-s001.pdf]

cells MDPI

Supplementary Material

S1. Protein expression analysis

Cell homogenates were prepared by ultrasonication in a buffer containing detergents and proteases and phosphatases inhibitors (Complete EDTA-free protease inhibitor cocktail and Phos-STOP phosphatase inhibitor cocktail, Roche Diagnostics, Milan, Italy). The detection of Parkin and CSA (tagged to CSA with HA) were separated on NuPAGE 4-12% precast polyacrylamide gels (Thermo Fischer Scientific, Carlsbad, US) and analyzed by western blotting with the following antibodies: mouse monoclonal anti-parkin (Sigma-Aldrich, Saint Louis, US), mouse monoclonal anti-HA (Sigma-Aldrich, Saint Louis, US). The detection of CSB was obtained on NuPAGE 3-8% precast polyacrylamide gels (Thermo Fischer Scientific, Carlsbad, US) and analyzed by western blotting with the rabbit polyclonal antibody (Bethil Laboratories). Immunocomplexes were revealed by using a peroxidase-conjugated secondary antibody (Biorad, CA, US). Quantitation of protein bands was performed by using Chemidoc (Biorad, CA, US). (See Figure S1)

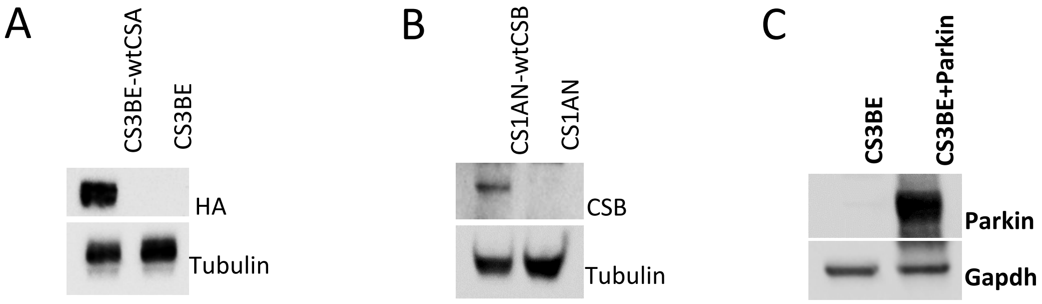

Figure S1. Characterization of cell lines by western blotting. (A) CS3BE-wtCSA expressing CSA protein tagged with HA and CS3BE defective cells with no expression of CSA; (B) CS1AN-wtCSB expressing CSB protein and CS1AN defective cells with no expression of CSB; (C) CS3BE+Parkin cells with overexpression of Parkin.

S2. Materials: Reagents, Chemicals and Enzymes

Nuclease P1 from Penicillium citrinum, phosphodiesterase I and II, alkaline phosphatase from bovine intestinal mucosa, DNase I and DNase II, benzonase 99%, BHT, deferoxamine mesylate and pentostatin were purchased from Sigma-Aldrich (Steinheim, Germany). RNase T1 was from Thermo Fisher Scientific (Waltham, MA, USA) and RNase A from Roche Diagnostic GmbH, (Mannheim, Germany). 2'-Deoxyadenosine monohydrate and 2'-deoxyguanosine monohydrate were purchased from Berry & Associates Inc. (Dexter, NY, USA). Isotopic labeled internal standards of 5′*R*-cdA, 5′*S*-cdA, 5′*R*-cdG, 5′*S*-cdG, 8-oxo-dA and 8-oxo-dG were prepared according to the previously reported procedures [1]. Solvents (HPLC-grade) were purchased from Fisher Scientific (Waltham, MA, USA). The 3kDa cut-off filters were obtained from Millipore (Bedford, OH, USA).

1. Terzidis, M.A.; Chatgilialoglu, C. An ameliorative protocol for the quantification of purine 5′,8-cyclo-2′-deoxynucleosides in oxidized DNA. *Front. Chem.* **2015**, 3, 47.

Table S1. A list of MRM transitions employed for the quantifications of the six oxidatively induced DNA lesions and their corresponding stable isotope-labeled standards.

Lesions	Precursor Ion m/z	Product Ion m/z	Collision Energy (V)
5'*R*-cdA	250	164	14
[$^{15}N_5$]-5'*R*-cdA	255	169	14
5'*S*-cdA	250	164	16
[$^{15}N_5$]- 5'*S*-cdA	255	169	16
5'*R*-cdG	266	180	18
[$^{15}N_5$]- 5'*R*-cdG	271	185	18
5'*S*-cdG	266	180	16
[$^{15}N_5$]- 5'*S*-cdG	271	185	16
8-oxo-dA	268	152	19
[$^{15}N_5$]- 8-oxo-dA	273	157	19
8-oxo-dG	284	168	18
[$^{15}N_5$]- 8-oxo-dG	289	173	18

Table S2. The levels (lesions/10^6 Nu) of 5'*R*-cdG, 5'*S*-cdG, 5'*R*-cdA, 5'*S*-cdA, 8-oxo-dG and 8-oxo-dA in DNA samples isolated from CS1AN-wtCSB and CS1AN cells in hyperoxic, physioxic and hypoxic conditions.[a.]

Sample	Conditions	5'R-cdG	5'R-cdA	5'S-cdG	5'S-cdA	8-oxo-dG	8-oxo-dA
CS1AN-wtCSB	hyperoxia	0.525±0.053	0.628±0.015	0.908±0.068	0.223±0.026	2.678±0.330	0.572±0.028
CS1AN-wtCSB	physioxia	0.591±0.120	0.731±0.045	0.975±0.085	0.257±0.028	3.149±0.203	0.819±0.029
CS1AN-wtCSB	hypoxia	0.742±0.226	0.799±0.168	1.317±0.252	0.292±0.027	3.307±0.427	0.722±0.088
CS1AN	hyperoxia	0.543±0.037	0.670±0.036	0.919±0.039	0.233±0.020	3.755±0.119	0.618±0.094
CS1AN	physioxia	0.601±0.071	0.754±0.054	1.016±0.074	0.280±0.004	4.133±0.309	1.007±0.056
CS1AN	hypoxia	0.870±0.104	1.033±0.128	1.521±0.139	0.353±0.032	4.836±0.770	1.137±0.205

[a] Mean values ± standard deviation of the three independent samples measurements.

Table S3. The levels (lesions/10^6 Nu) of 5'*R*-cdG, 5'*S*-cdG, 5'*R*-cdA, 5'*S*-cdA, 8-oxo-dG and 8-oxo-dA in DNA samples isolated from CS3BE-wtCSA and CS3BE cells in hyperoxic, physioxic and hypoxic conditions.

Sample	Conditions	5'R-cdG	5'R-cdA	5'S-cdG	5'S-cdA	8-oxo-dG	8-oxo-dA
CS3BE-wtCSA	hyperoxia	0.387 ± 0.011	0.572 ± 0.026	0.867 ± 0.017	0.231 ± 0.036	2.082 ± 0.144	0.549 ± 0.056
CS3BE-wtCSA	physioxia	0.409 ± 0.003	0.574 ± 0.035	0.885 ± 0.081	0.258 ± 0.012	2.748 ± 0.271	0.751 ± 0.054
CS3BE-wtCSA	hypoxia	0.405 ± 0.047	0.595 ± 0.036	0.901 ± 0.205	0.300 ± 0.052	2.698 ± 0.334	0.666 ± 0.076
CS3BE	hyperoxia	0.419 ± 0.015	0.664 ± 0.024	0.925 ± 0.053	0.196 ± 0.014	2.649 ± 0.303	0.749 ± 0.074
CS3BE	physioxia	0.481 ± 0.057	0.709 ± 0.076	1.032 ± 0.105	0.263 ± 0.020	3.168 ± 0.512	1.080 ± 0.048
CS3BE	hypoxia	0.843 ± 0.162	1.261 ± 0.216	1.226 ± 0.114	0.411 ± 0.038	4.427 ± 0.543	1.420 ± 0.279

[a] Mean values ± standard deviation of the three independent samples measurements.

Table S4. Values of t-Test (Two-Sample Assuming Unequal Variances) by comparing the means of lesions in DNA samples isolated from CS1AN-wtCSB and CS1AN cells in each condition.

Samples	Conditions	5'*R*-cdG	5'*R*-cdA	5'*S*-cdG	5'*S*-cdA	8-oxo-dG	8-oxo-dA
CS1AN-wtCSB vs CS1AN	Hyperoxia	0.754	0.142	0.704	0.654	0.053	0.396
CS1AN-wtCSB vs CS1AN	Physioxia	0.921	0.689	0.522	0.260	0.005**	0.011*
CS1AN-wtCSB vs CS1AN	Hypoxia	0.272	0.027*	0.273	0.003**	0.018*	0.028*
CS1AN-wtCSB vs CS1AN-wtCSB	Hyperoxia vs Physioxia	0.451	0.081	0.518	0.290	0.093	0.017*
CS1ANvs CS1AN	Hyperoxia vs Physioxia	0.116	0.106	0.091	0.073	0.226	0.044*
CS1AN-wtCSB vs CS1AN-wtCSB	Hyperoxia vs Hypoxia	0.649	0.248	0.005**	0.559	0.274	0.123
CS1ANvs CS1AN	Hyperoxia vs Hypoxia	0.031	0.058	0.012*	0.184	0.010*	0.333
CS1AN-wtCSB vs CS1AN-wtCSB	Physioxia vs Hypoxia	0.982	0.521	0.051	0.175	0.602	0.156
CS1ANvs CS1AN	Physioxia vs Hypoxia	0.089	0.063	0.032*	0.676	0.235	0.422

Statistically significant samples: * <0.05 , **<0.01.

Table 5. Values of t-Test (Two-Sample Assuming Unequal Variances) by comparing the means of lesions in DNA samples isolated from CS3BE-wtCSA and CS3BE cells in each condition.

Samples	Conditions	5'*R*-cdG	5'*R*-cdA	5'*S*-cdG	5'*S*-cdA	8-oxo-dG	8-oxo-dA
CS3BE-wtCSA vs CS3BE	Hyperoxia	0.037*	0.087	0.210	0.351	0.151	0.040*
CS3BE-wtCSA vs CS3BE	Physioxia	0.172	0.105	0.102	0.707	0.374	0.003**
CS3BE-wtCSA vs CS3BE	Hypoxia	0.047*	0.045*	0.049*	0.166	0.044*	0.053
CS3BE-wtCSA vs CS3BE-wtCSA	Hyperoxia vs Physioxia	0.098	0.958	0.759	0.408	0.104	0.047*
CS3BE vs CS3BE	Hyperoxia vs Physiox.	0.134	0.272	0.348	0.058	0.134	0.042*
CS3BE-wtCSA vs CS3BE-wtCSA	Hyperoxia vs Hypoxia	0.561	0.413	0.802	0.063	0.044*	0.231
CS3BE vs CS3BE	Hyperoxia vs Hypoxia	0.127	0.038*	0.072	0.006**	0.028*	0.081
CS3BE-wtCSA vs CS3BE-wtCSA	Physioxia vs Hypoxia	0.904	0.645	0.839	0.369	0.885	0.352
CS3BE vs CS3BE	Physioxia vs Hypoxia	0.284	0.033*	0.164	0.02*	0.134	0.126

Statistically significant samples: * <0.05 , **<0.01.

Table S6. 5'*R*/5'*S* ratio of cPu lesions in DNA isolated from CS1AN-wtCSB and CS1AN cells and CS3BE-wtCSA and CS3BE cells in hyperoxic, physioxic and hypoxic conditions.

Samples	Conditions	cdG 5′*R*/5′*S*	cdA 5′*R*/5′*S*
**CS1AN-wtCSB**	Hyperoxia	0.58 ± 0.03	2.84 ± 0.34
CS1AN-wtCSB	Physioxia	0.61 ± 0.14	2.88 ± 0.50
CS1AN-wtCSB	Hypoxia	0.55 ± 0.08	2.73 ± 0.51
CS1AN	Hyperoxia	0.59 ± 0.05	2.89 ± 0.35
CS1AN	Physioxia	0.59 ± 0.05	2.69 ± 0.23
CS1AN	Hypoxia	0.57 ± 0.05	2.93 ± 0.26
CS3BE-wtCSA	Hyperoxia	0.45 ± 0.02	2.53 ± 0.49
CS3BE-wtCSA	Physioxia	0.46 ± 0.04	2.22 ± 0.06
CS3BE-wtCSA	Hypoxia	0.47 ± 0.13	2.01 ± 0.28
CS3BE	Hyperoxia	0.45 ± 0.02	3.40 ± 0.36
CS3BE	Physioxia	0.47 ± 0.11	2.70 ± 0.19
CS3BE	Hypoxia	0.69 ± 0.13	3.07 ± 0.47

Table S7. Total amount of cPu and 8-oxo-Pu lesions in DNA isolated from CS1ANwtCS-B and CS1AN cells in hyperoxic, physioxic and hypoxic conditions.

Samples	Conditions	cPu	8-oxo-Pu
CS1ANwtCS-B	Hyperoxia	2.284 ± 0.139	3.249 ± 0.304
CS1ANwtCS-B	Physioxia	2.554 ± 0.137	3.968 ± 0.223
CS1ANwtCS-B	Hypoxia	3.150 ± 0.655	4.028 ± 0.440
CS1AN	Hyperoxia	2.367 ± 0.062	4.373 ± 0.186
CS1AN	Physioxia	2.651 ± 0.182	5.140 ± 0.358
CS1AN	Hypoxia	3.776 ± 0.371	5.974 ± 0.708

Table S8. Total amount of cPu and 8-oxo-Pu lesions in DNA isolated from CS3BE-wtCSA and CS3BE cells in hyperoxic, physioxic and hypoxic conditions.

Samples	Conditions	cPu	8-oxo-Pu
CS3BE-wtCSA	Hyperoxia	2.056 ± 0.030	2.632 ± 0.122
CS3BE-wtCSA	Physioxia	2.125 ± 0.128	3.499 ± 0.299
CS3BE-wtCSA	Hypoxia	2.202 ± 0.121	3.364 ± 0.397
CS3BE	Hyperoxia	2.204 ± 0.055	3.398 ± 0.240
CS3BE	Physioxia	2.485 ± 0.136	4.247 ± 0.559
CS3BE	Hypoxia	3.741 ± 0.453	5.847 ± 0.543

Table S9. Values of t-Test (Two-Sample Assuming Unequal Variances) by comparing the sum of cPu and 8-oxo-Pu lesions in DNA isolated from CS1ANwtCS-B and CS1AN cells in each condition.

Samples	Conditions	cPu	8-oxo-Pu
CS1ANwtCS-B vs CS1AN	Hyperoxia	0.532	0.054
CS1ANwtCS-B vs CS1AN	Physioxia	0.585	0.006**
CS1ANwtCS-B vs CS1AN	Hypoxia	0.138	0.007**
CS1ANwtCS-B	Hyperoxia vs Physioxia	0.178	0.035*
CS1ANwtCS-B	Hyperoxia vs Hypoxia	0.153	0.027*
CS1ANwtCS-B	Physioxia vs Hypoxia	0.319	0.714
CS1AN	Hyperoxia vs Physioxia	0.054	0.127
CS1AN	Hyperoxia vs Hypoxia	0.023*	0.090
CS1AN	Physioxia vs Hypoxia	0.043*	0.082

Statistically significant samples: * <0.05, ** <0.01.

Table S10. Values of t-Test (Two-Sample Assuming Unequal Variances) by comparing the sum of cPu and 8-oxo-Pu lesions in DNA isolated from CS3BE-wtCSA and CS3BE cells in each condition.

Samples	Conditions	cPu	8-oxo-Pu
CS3BE-wtCSA vs CS3BE	Hyperoxia	0.035*	0.067
CS3BE-wtCSA vs CS3BE	Physioxia	0.051	0.189
CS3BE-wtCSA vs CS3BE	Hypoxia	0.016*	0.041*
CS3BE-wtCSA	Hyperoxia vs Physioxia	0.519	0.064
CS3BE-wtCSA	Hyperoxia vs Hypoxia	0.197	0.044*
CS3BE-wtCSA	Physioxia vs Hypoxia	0.249	0.751
CS3BE	Hyperoxia vs Physioxia	0.117	0.084
CS3BE	Hyperoxia vs Hypoxia	0.034*	0.007**
CS3BE	Physioxia vs Hypoxia	0.028*	0.065

Statistically significant samples: * <0.05, ** <0.01.

Table S11. The levels (lesions/10^6 Nu) of 5'*R*-cdG, 5'*S*-cdG, 5'*R*-cdA, 5'*S*-cdA, 8-oxo-dG and 8-oxo-dA in DNA samples isolated from CS3BE-wtCSA, CS3BE and CS3BE+Parkin cells. The numbers in the boxes represent the mean value (± standard deviation) of DNA lesions levels from the measurement of three samples.

Sample	5'*R*-cdG	5'*R*-cdA	5'*S*-cdG	5'*S*-cdA	8-oxo-dG	8-oxo-dA
CS3BE-wtCSA	0.486 ± 0.070	0.737 ± 0.075	0.829 ± 0.029	0.289 ± 0.010	3.092 ± 0.157	0.825 ± 0.054
CS3BE	0.500 ± 0.137	0.834 ± 0.164	0.961 ± 0.133	0.311 ± 0.033	3.854 ± 0.656	0.984 ± 0.145
CS3BE+Parkin	0.462 ± 0.029	0.785 ± 0.088	0.840 ± 0.099	0.323 ± 0.041	2.942 ± 0.330	0.756 ± 0.057

Table S12. Values of *t*-Test (Two-Sample Assuming Unequal Variances) by comparing the means of lesions in each condition.

**Samples**	**5'*R*-cdG**	**5'*R*-cdA**	**5'*S*-cdG**	**5'*S*-cdA**	**8-oxo-dG**	**8-oxo-dA**
CS3BE-wtCSA vs CS3BE	0.908	0.494	0.293	0.255	0.244	0.225
CS3BE-wtCSA vs CS3BE+Parkin	0.574	0.601	0.889	0.293	0.646	0.390
CS3BE vs CS3BE+Parkin	0.730	0.377	0.179	0.681	0.058	0.105

Table S13. Total amount of cPu and 8-oxo-Pu lesions in DNA isolated from CS3BE-wtCSA, CS3BE and CS3BE+Parkin cells. The numbers in the boxes represent the mean value (± standard deviation) of DNA lesions levels from the measurement of three samples.

**Sample**	**cPu**	**8-oxo-Pu**
CS3BE-wtCSA	2.341 ± 0.029	3.917 ± 0.199
CS3BE	2.606 ± 0.353	4.838 ± 0.537
CS3BE+Parkin	2.409 ± 0.089	3.698 ± 0.371
